# Printed Receive Coils with High Acoustic Transparency for Magnetic Resonance Guided Focused Ultrasound

**DOI:** 10.1038/s41598-018-21687-1

**Published:** 2018-02-21

**Authors:** Joseph Corea, Patrick Ye, Dongjin Seo, Kim Butts-Pauly, Ana Claudia Arias, Michael Lustig

**Affiliations:** 1 Electrical Engineering and Computer Sciences, University of California, Berkeley, CA 94720 USA; 2 Radiology, Stanford University, Stanford, CA 94305 USA

## Abstract

In magnetic resonance guided focused ultrasound (MRgFUS) therapy sound waves are focused through the body to selectively ablate difficult to access lesions and tissues. A magnetic resonance imaging (MRI) scanner non-invasively tracks the temperature increase throughout the tissue to guide the therapy. In clinical MRI, tightly fitted hardware comprised of multichannel coil arrays are required to capture high quality images at high spatiotemporal resolution. Ablating tissue requires a clear path for acoustic energy to travel but current array materials scatter and attenuate acoustic energy. As a result coil arrays are placed outside of the transducer, clear of the beam path, compromising imaging speed, resolution, and temperature accuracy of the scan. Here we show that when coil arrays are fabricated by additive manufacturing (i.e., printing), they exhibit acoustic transparency as high as 89.5%. This allows the coils to be placed in the beam path increasing the image signal to noise ratio (SNR) five-fold in phantoms and volunteers. We also characterize printed coil materials properties over time when submerged in the water required for acoustic coupling. These arrays offer high SNR and acceleration capabilities, which can address current challenges in treating head and abdominal tumors allowing MRgFUS to give patients better outcomes.

## Introduction

The precise heating of deep lying tissue has already shown to be effective in selectively ablating lesions, opening the blood brain barrier, and in stimulating specific nerves^1–6^. Magnetic resonance guided focused ultrasound (MRgFUS) has been successfully applied to treat uterine fibroids^3,7^ and soft tissue tumors^8^. It can also substantially reduce the pain from bone cancer metastasis^9^ and the shaking from essential tremor^10^. While traditional surgery, radiosurgery, or deep brain stimulation are available to treat these conditions^11^, MRgFUS offers similar or better outcomes without the major complications that can arise from exposing the patient to high doses of radiation or relying on an invasive surgery^3,7,9,10^. However, the current image quality and temporal acceleration used to guide the surgery has been a limiting factor in the widespread implementation of this technique, particularly for brain and abdominal applications.

**Figure 1:**
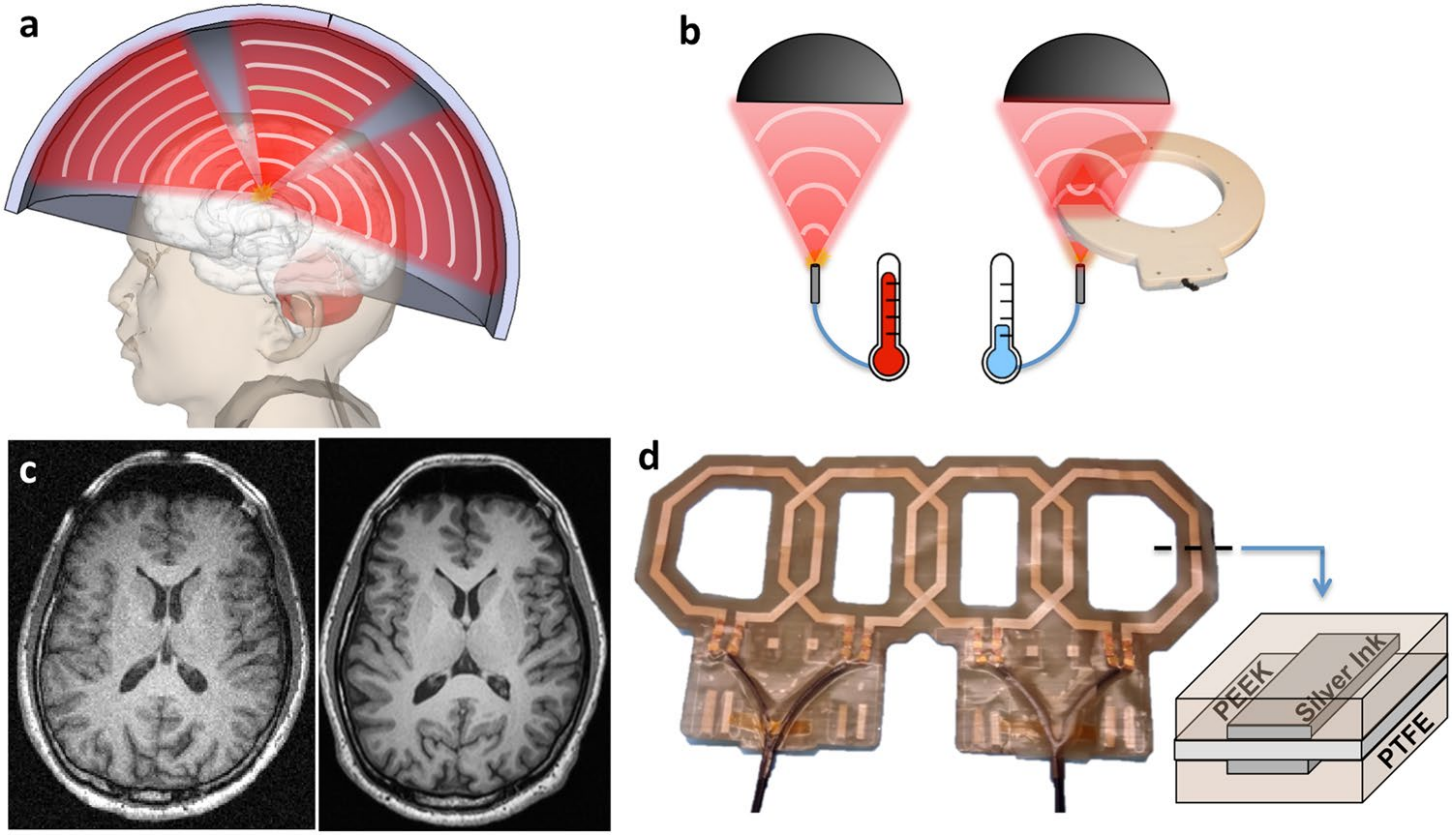
MRgFUS requires receive coils to be close to the patient and transparent to acoustic energy. (**a**) Illustration of patient positioned inside a brain transducer. Ultrasonic energy (red) is passed through water to heat deep lying tissue. (**b**) Illustration of acoustic energy (red) focusing onto thermal probe producing heat with and without a conventionally constructed surface coil present. (**c**) Axial MRI scan of human brain using low SNR body coil (left) and higher SNR surface coil (right) using the same sequence parameters. (**d**) Photograph of thin acoustically transparent surface array fabricated layer-by-layer using screen printing. Inset shows the cross section of the array detailing the materials used for construction. Note that the apparent amber color of the silver conductors is caused by the brown color of the PTFE surface treatment.

Water is used to efficiently transfer ultrasonic energy from the acoustic transducer to the patient. Figure 1A illustrates how sound waves (i.e., ultrasonic energy) are focused through water and tissue to an ellipsoidal point deep inside a patient’s body. The size of the focal point depends on the geometry of the transducer, but a typical spot size for a clinical system is approximately 1.3 mm in diameter and 2.6 mm long. This small focal point raises the temperature locally without damaging the surrounding tissue. The system estimates the change in temperature by comparing the phase difference between a baseline MR image to one acquired during heating^2^. This heat map enables interventional radiologists to accurately plan the therapy and to move the focal point to the desired area^12^.

High signal to noise ratio (SNR) is required to quickly produce high resolution images with accurate temperature estimation^2^. In traditional MRI, high SNR images are acquired using multiple channel surface coil arrays that are placed in close contact with the patient^13,14^. Surface coil arrays can accelerate the image acquisition using parallel imaging^15^ by exploiting the spatial sensitivities of coils. In turn, faster acquisitions reduce artifacts from patient motion.

During therapy, the transducer is moved around the patient while changing the focus of the transducer to sweep the relatively small focal point throughout a larger volume. If a surface array is placed in front of the transducer, the transducer would pass acoustic energy directly through different parts of a tightly fitting surface coil as it is moved. This would cause unpredictable attenuation and scattering of the focal point^16^. Figure 1B shows how ultrasonic energy easily scatters and attenuates in traditional surface coil materials, drastically reducing the temperature at the focal point and reflecting energy elsewhere. The loss from the surface coil components is significant due to their thickness and to the large difference in acoustic impedance compared to water^17^. As a result, large volume coils or non-fitting surface coils that are positioned out of the way of the beam are used to image during therapy. These non-fitting coils offer lower SNR at the treatment site and are often unable to take advantage of image acceleration techniques. This significantly degrades the temporal and spatial resolution of the temperature estimation and leads to low quality temperature maps and images with motion artifacts. Additionally, the anatomy images used to plan surgery lack the resolution needed to see critical features, such as nerves, requiring patients to monitor extremity sensation while enduring uncomfortable and painful procedures without general anesthesia^18,19^. *In-situ* tracking of tissue necrosis with diffusion-weighted images is also severely limited by the low SNR of current MRgFUS coils compared to the SNR offered by traditional multi-channel coil arrays^20,21^. To illustrate the SNR improvement that can be gained by using surface coils, examples of a low SNR image taken with a body coil and a high SNR image from a surface coil array are shown in Fig. 1C. As Fig. 1C shows, the image taken with the body coil has more noise making it harder to see fine features compared to the image acquired with the surface array.

It has been reported that higher SNR in MRgFUS is achieved by introducing novel receive coils such as those made for head and breast imaging^22–24^. However, these reports have focused on the implementation of existing materials, positioning materials out of the way of the transducer elements to avoid acoustic scattering from coil components. Increased SNR is also achieved by adding a thin dipole antenna that does not significantly impact acoustic attenuation to the design^25^. However, these approaches add considerable constrains to the design and implementation of the coil array. Other techniques that do not rely on close proximity of the probe, such as traveling-wave MRI^26^, have poor SNR and are not suited to image on 1.5 and 3 T scanners where MRgFUS procedures currently take place.

A surface coil array that is transparent to acoustic energy would drastically increase image quality and temperature estimation. One way to fabricate an acoustically transparent coil is to use very thin polymer-based materials and solution processed conductors. These materials can be selected to have acoustic properties close to that of water reducing the amount of interaction with the acoustic energy. We have shown in previous work that it is possible to create such coils with screen-printed conductive inks on thin plastic substrates^27,28^. A surface coil is a resonant loop of wire tuned to resonate at the Larmor frequency of the scanner using in-series capacitors. To fabricate these coils, solution processed conductors are selectively deposited in a loop on a flexible plastic substrate with tuning capacitors^27–30^. Figure 1D shows an example of a surface array made in this way highlighting how the conductive traces sandwich the plastic substrate to form very thin capacitors. The capacitance depends on the amount of overlap, substrate material, and substrate thickness. The printing and ink drying processes uses temperatures between 80–140 °C, allowing for a wide variety of common plastics to be used for coil fabrication^28^. In this work we detail the acoustic properties of the printed and traditional coil materials, compare the imaging performance of screen-printed coil arrays to the body coil used during MRgFUS treatments, and show the performance of screen-printed arrays in fibroid and head MRgFUS systems. Our method for fabricating coils addresses the current limitations with existing coil materials and enables high image SNR while maintaining high acoustic transparency.

## Results

### Water Stability of Printed Coil Materials

For a coil to be useful in a clinical therapy, it must be able to maintain the same electrical characteristics while it is exposed to water for the length of the procedure. It is common for MRgFUS treatments to last 3 hours or more^31^ therefore to meet this requirement, the coils need to be fabricated with materials that do not suffer property changes over time when submerged. If the materials used in coil construction are affected by water, the coil function can be significantly compromised as the conductor or dielectric degrade.

**Figure 2:**
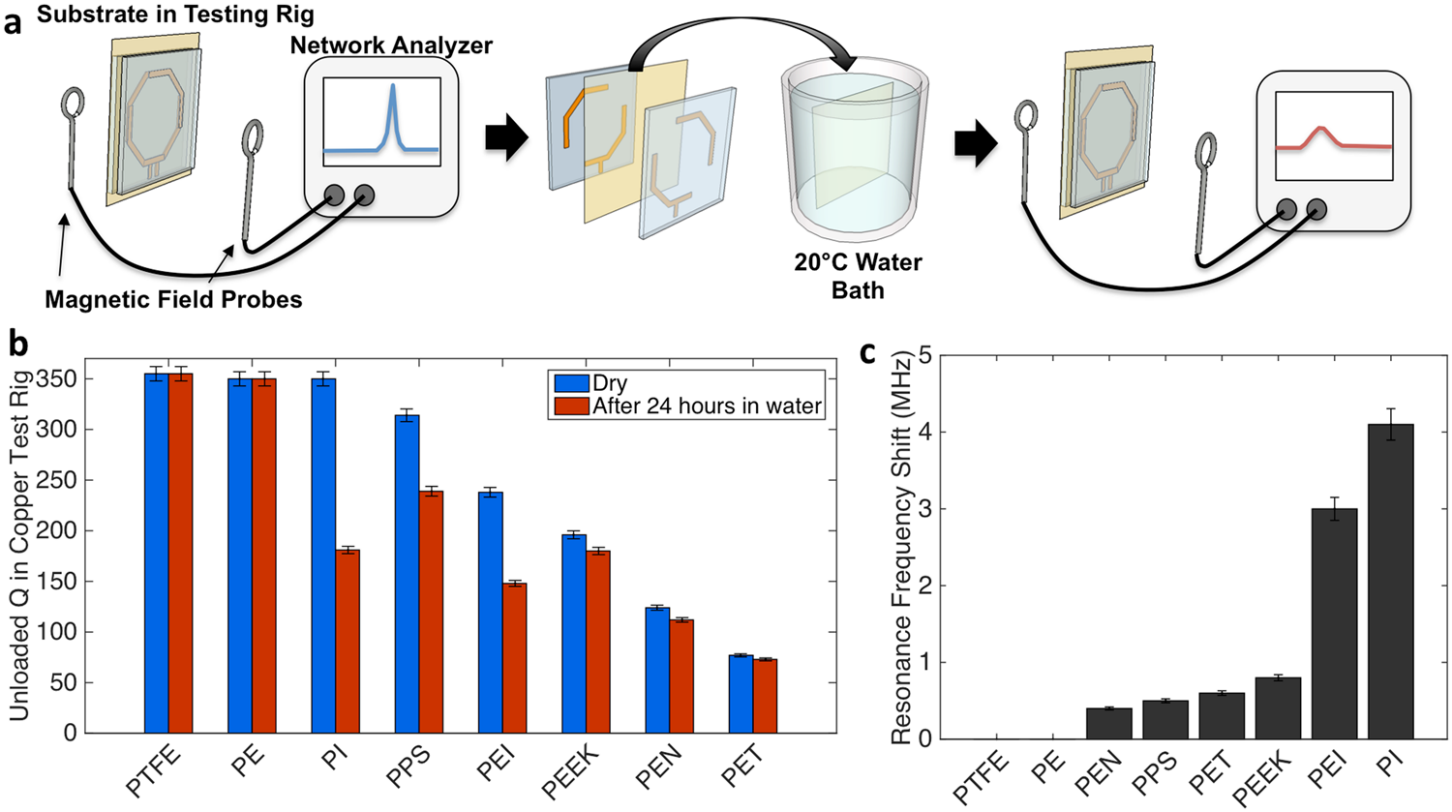
Substrate behavior with exposure to water. (**a**) Illustration showing coil how test film (yellow) is clamped into copper and acrylic testing rig to measure coil Q and resonant frequency. Film is removed and submerged in water for 24 hours. Finally coil film is removed from water and Q and resonant frequency are re-measured. (**b**) Substrate Q_Unloaded_ before and after water immersion in test rig. Error bars show range of Q measurements. (**c**) Resonant frequency shift for several test substrates after they are immersed in water for 24 hours. Error bars show range of frequency measured.

To investigate suitable materials for coil fabrication, we characterize several plastic substrates for water absorption and electrical quality. While most plastics have been characterized in terms of water absorption and loss, it is uncommon to report loss data at frequencies common to MRI. Furthermore, any moisture dependence of dielectric quality is rarely captured in standard testing. To characterize materials for use in MRgFUS coils, the substrates are placed in a copper/acrylic testing rig to simulate the final coil structure described in our previous work^28^. Figure 2A shows how the quality factor (Q) of the resonator is measured before and after the plastic substrate is submerged in 20 °C deionized water for 24 hours. Q is a quantity that describes the amount of reactance in comparison to the resistance in a resonant circuit, with higher values indicating less loss. From the relative changes in Q and resonant frequency, we are able to compare coil materials to determine a substrate that presents both high Q and insensitivity to water. Barrier materials provide some protection against water impinging on the substrate and changing the electrical properties of the coil. However, water can advance into the coil along the edges of the substrate and through the adhesive used to bond the film to the substrate therefore the use of a water resistant substrate is preferred.

Here, polytetrafluoroethylene (PTFE), polyethylene (PE), polyimide (PI), polyphenylene sulfide (PPS), polyetherimide (PEI), polyether ether ketone (PEEK), polyethylene naphthalate (PEN) and polyethylene terephthalate (PET) are evaluated. Figure 2B shows the change in the Q value while Fig. 2C shows the change in the resonant frequency that the coils experienced before and after submersion in water for 24 hours. Any change in Q before and after submersion is more important than the maximum Q value for any particular substrate. Material properties that vary with exposure to water make tuning the coil challenging as any absorbed water changes the coil tuning which significantly degrades image SNR. For example, PI, PPS, and PEI show higher Q than PEEK, but after submersion in water the resonant frequency and Q significantly change. The shift in the coil tuning is due to the large difference in dielectric constants between plastics (ε_r_ ≈ 2–4) and water (ε_r_ = 80 at 20 °C), therefore even a small amount of absorbed water has a large impact on the resonant frequency. Other substrates such as PE and PTFE show high Q values with very small shift, but are not suitable for the printing process due to poor adhesion of the conductive ink and are easily deformed by mechanical stress. A PEEK substrate is selected as the most appropriate material to fabricate MRgFUS coils with due to its high Q, low water absorption, and conductive ink compatibility.

DuPont 5064 H silver ink is investigated for the conductive portions of the coil based on its previous use in printed MRI coils^28^. After 24 hours of water submersion, the samples made of the DuPont 5064 H silver ink did not experience any significant change in resistivity; showing resistivity of 16 ± 2 μohm-cm before and after. Furthermore, the surface roughness of the ink did not change, maintaining a root mean squared (RMS) surface roughness of 1.3 ± 0.2 μm both times.

In this study the coil is placed in the water bath, however in some transducer systems it may be more desirable to place the coil in the oil bath that surrounds some transducers. The differences in the oil surface energy, dielectric constant, and solubility in the substrates compared to water make it difficult to extrapolate the findings here and predict coil performance in such systems, but would be a good candidate for future study.

### Acoustic Properties of Printed Coils

**Figure 3:**
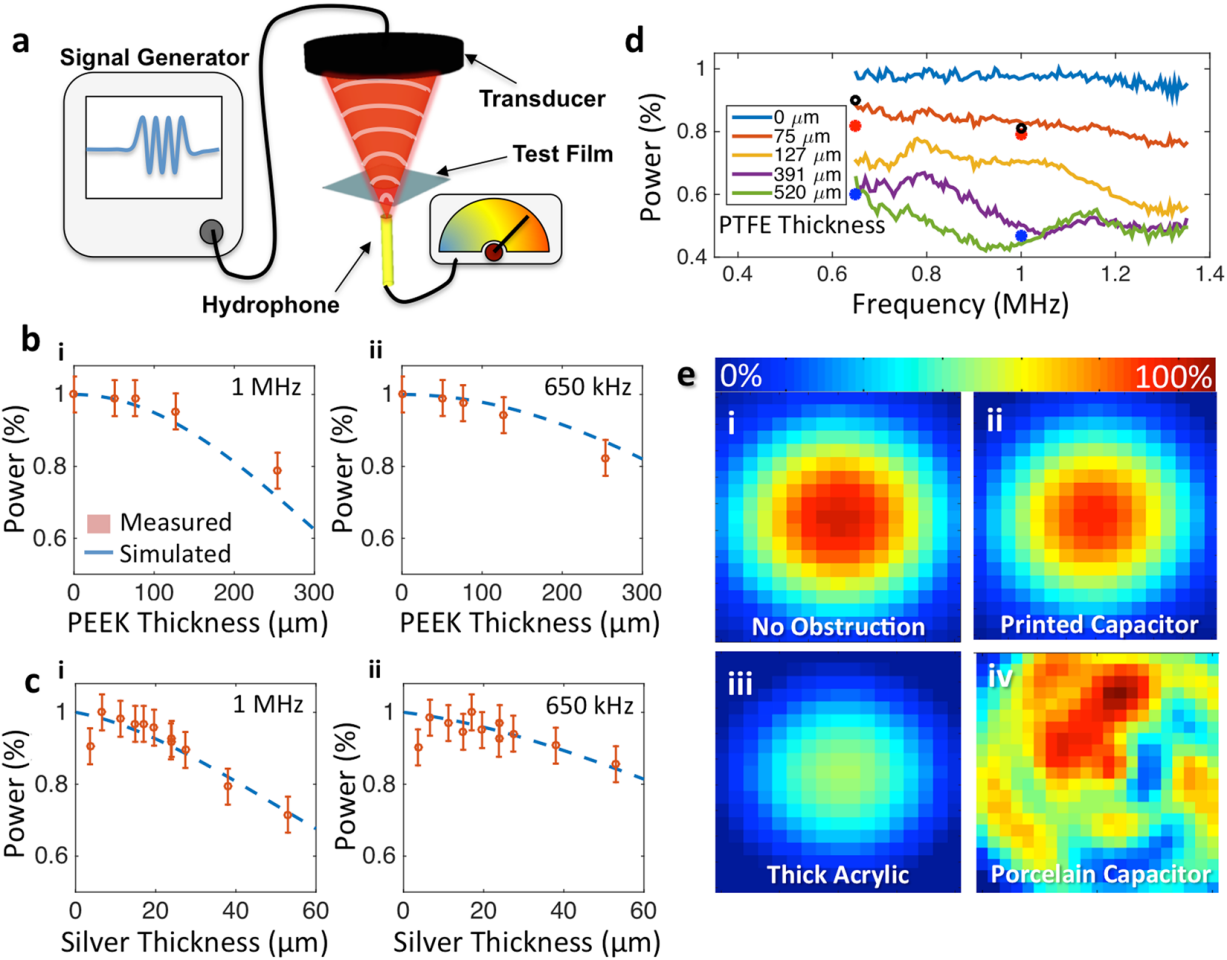
Ultrasonic power transmission properties of substrates. (**a**) Illustration showing substrate (blue) placement during sonication testing. Transducer (black) is driven at different frequencies with acoustic pressure measured by a hydrophone (yellow). Transducer, substrate, and hydrophone are submerged in degassed, deionized, water. (**b**) Relative acoustic power for several thicknesses of PEEK plastic at (i) 1 MHz and (ii) 650 kHz. Dotted blue line shows attenuation estimation from acoustic model described in the supporting material. Error bars show standard deviation. (**c**) Relative acoustic power for several thicknesses of DuPont 5064 H silver ink on 76 μm thick PEEK plastic at (i) 1 MHz and (ii) 650 kHz. Dotted blue line shows the attenuation estimation from the acoustic model. Error bars show standard deviation. (**d**) Relative acoustic power transmitted through several thicknesses of PTFE encapsulation on both sides of 76 μm thick PEEK plastic. Black dots show relative intensity from a printed capacitor with PTFE encapsulation. Red dots indicate transmission from a flexible circuit board capacitor made from 9 μm of copper on 50 μm of polyimide. Blue dots indicate transmission from a flexible circuit board capacitor made from 35 μm of copper on 50 μm of polyimide. (**e**) Spot profiles for transducer focal point over as thy hydrophone is scanned over a 4 × 4 mm area showing relative intensity with (i) no obstruction, (ii) printed coil capacitor in beam path, (iii) 2 mm acrylic plastic, and (iv) traditionally used porcelain tuning capacitor in beam path.

The coil materials must also transmit a high percentage of incident acoustic energy without distortion. Local surface burns, damage to the transducer, and low focal heating occur if the coils reflect or attenuate a significant amount of the acoustic energy. To characterize the films, a transducer passes acoustic power through test films to a hydrophone that records the acoustic intensity, as illustrated in Fig. 3A.

The acoustic absorption of PEEK is characterized in the thickness range of 50 μm to 254 μm to determine the optimal thickness. All film thicknesses are within 10% of the reported values. Figure 3B shows the relative acoustic power measured from several samples of PEEK at 650 kHz and 1 MHz – frequencies common to head and body MRgFUS, respectively. It can be seen that the thinnest films of PEEK provide the least amount of attenuation; however, thinner films are more difficult to process as they are more susceptible to mechanical damage. As a result, we chose a PEEK film thickness of 76 μm to maintain acoustic transparency, handling robustness, and ease of processing

The acoustic properties of solution-processed materials are not commonly available. To determine the acoustic impedance of the conductive silver ink we transmitted acoustic power though several thicknesses (3–56 μm) of the silver film deposited on the 76 μm of PEEK film. Figure 3C shows the relative acoustic power measured through several samples of silver ink on PEEK film at 650 and 1 MHz. Also shown in Fig. 3B,C, are the results from simulations using an acoustic model. The measured values of transmitted acoustic power are in agreement with the predicted transmitted power, suggesting that the printed silver films are attenuating the acoustic energy mainly by transmission and reflection interactions rather than by diffuse scattering or bulk attenuation. By fitting our data to the acoustic model we found that the DuPont 5064 H silver ink has an acoustic impedance of 15.6 ± 3.8 MRayls. This value is closer to that of water at 1.5 MRayls, when compared to commonly used copper at 44.6 MRayls or bulk silver at 38.0 MRayls^17,32,33^. This decreased acoustic impedance can be attributed to the composition of the ink, which is composed of a suspension of silver micro-flakes into polymer-based binders that remain in the film after the thermal curing process. The silver microflakes in the ink have an acoustic impedance similar to bulk silver while the polymer binders have a lower acoustic impedance, similar to most plastics. Combining the two gives acoustic properties in between the two constituent materials, like those shown in our measurement. The decreased acoustic impedance allows reduced reflections at any water, tissue, or plastic interface compared to commonly used conductors. If higher acoustic transparency were desired, the ink could be reformulated to increase the load of low acoustic impedance materials in the solution. There would be a trade off between conductivity and acoustic transparency. An in-depth study of the effects of ink formulation on film conductivity, adhesion, mechanical robustness, and printing characteristics would be required and is beyond the scope of the work presented here. Overall the acoustic properties of the commercially available silver ink make it well suited for use in the acoustically transparent coils.

To protect the patient from any DC bias that might exist on the coil, an electrically isolating film is deposited over the conductive traces. This film must be acoustically transparent in addition to providing high electrical breakdown strength. From the encapsulation testing described in the extended methods, we found that a PTFE film is the most appropriate for further characterization and optimization. Test films with 75, 127, 391, and 520 μm in thickness of PTFE were measured for transmission across a span of common MRgFUS frequencies. Figure 3D shows the percentage of power transmitted through the PTFE/PEEK/PTFE test film over a span of frequencies. The highest transmission across all frequencies is given by 76 μm of PTFE film on both sides of the 76 μm PEEK substrate. As a result, this stack is used for our coil construction.

The optimized material stack of a 76 μm thick PEEK substrate encapsulated in 76 μm of PTFE with 15 μm of the printed conductor is further characterized by comparing it to the traditional materials used in coil construction. Figure 3E shows the 2D acoustic power transmission profiles for our printed capacitor in addition to the traditionally used coil circuit and encapsulation materials. From these 2D acoustic pressure maps, we were not able to notice any significant distortion or scattering in the focal spot for the printed capacitor. The printed capacitor transmitted 80.5% of the acoustic power at 1 MHz and 89.5% at 650 kHz, in agreement with previous testing. These transmissions are much higher compared with the 51.4% and 62.5% obtained with the 2 mm thick acrylic. The beam shape is also preserved for both the acrylic and printed capacitors, but it is significantly scattered for the traditionally used porcelain capacitor on copper clad fiberglass reinforced circuit board.

To provide a comparison to a non-printed approach, two commonly available thin copper clad substrates are also evaluated by our hydrophone setup. Commercially available 9 μm copper on top of 50 μm polyimide (Pyralux AP 7156E) and 35 μm copper on top of 50 μm polyimide (Pyralux AP 9121 R) are both encapsulated in 76 μm of PTFE and characterized for comparison to our printed coil. The transmitted acoustic power for these films is shown in Fig. 3D and indicates that while the thinner copper passes 95% of the power compared to the printed coil, the printed coil outperforms the copper coil at both 650 kHz and 1 MHz. In addition to providing poorer acoustic transmission, the Pyralux substrates are made of materials that are sensitive to water. The copper conductors easily corrode and break down if left in water for extended periods of time. The polyimide substrate materials readily absorb water changing the electrical tuning of any coil made from it. For example, when the Pyralux substrate is exposed to water for 24 hours and measured in the Q-testing rig as the other substrates were, the Pyralux absorbed enough water to drop the Q from 356 to 232 and shift the resonant frequency 2.5 Mhz.

### Acoustically Transparent Coil Array Imaging Performance

**Figure 4:**
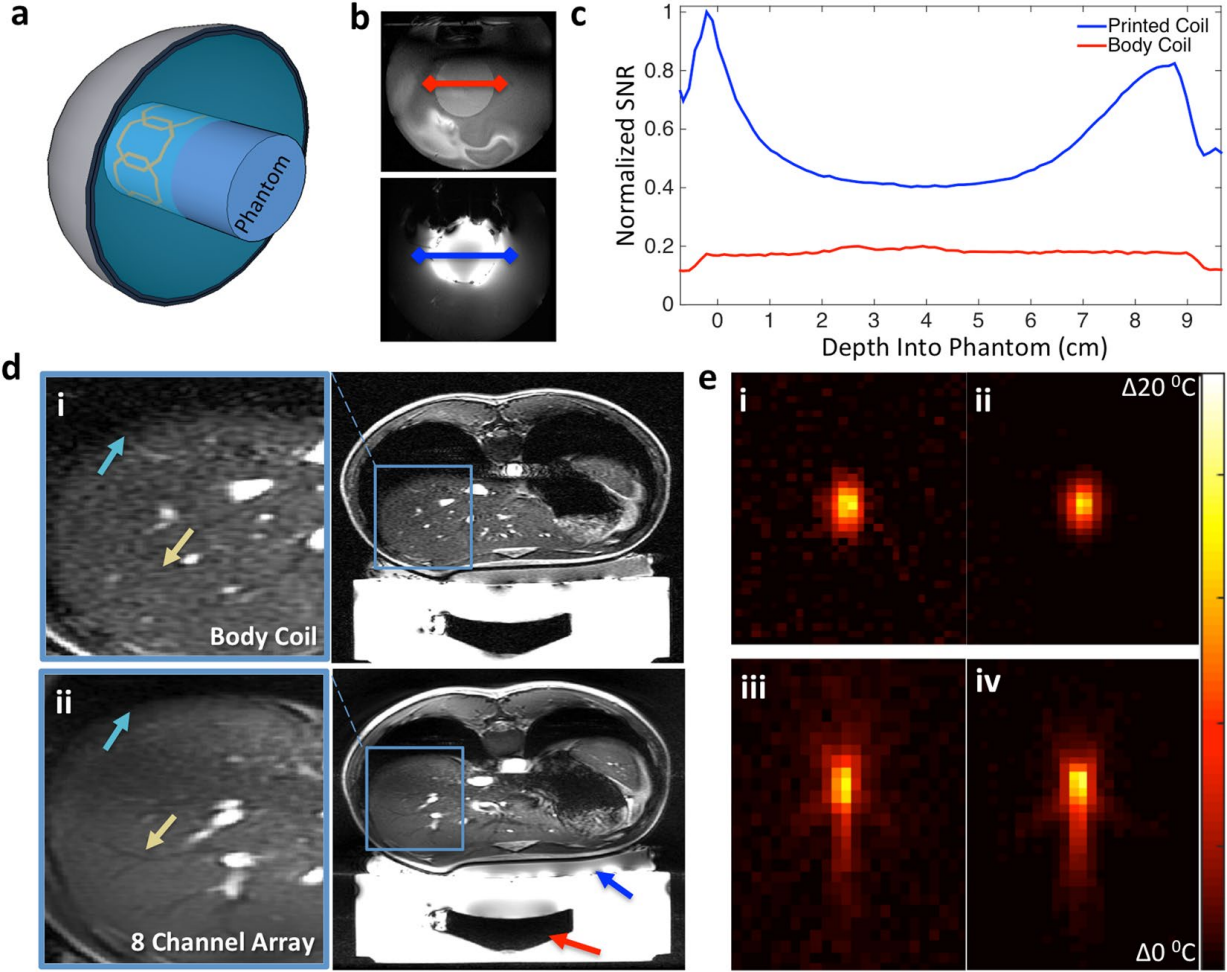
Phantom imaging and tracking in the head transducer. (**a**) Illustration of the experimental setup. Phantom is placed inside a head transducer with a 4-channel coil array wrapped around. The entire transducer is filled with water. (**b**) Axial slices of phantom in head array with red and blue lines showing location of SNR calculation acquired with 4-channel array. (**c**) SNR for 4-channel printed surface array compared to scanner equipped body coil. Magnitude is normalized to printed coil. (**d**) Abdominal images of volunteer using (i) low SNR body coil and (ii) high SNR 8-channel ultrasound transparent array. Teal and yellow arrows highlight liver/lung interface and veins in liver where image quality is increased from the array. Red arrow shows location of transducer, blue arrow indicates location of array. (**e**) Heat maps tracking sonication inside cylindrical gel phantom using axial scans of focal point using (i) body coil and (ii) 4-channel printed array. Coronal scans of focal point using (iii) body coil and (iv) 4-channel printed coil array. The standard deviation of the temperature estimation was ± 0.84 °C from images obtained with the body coil and ± 0.19 °C in images from the array.

Our coils must provide higher SNR than what is currently available in clinical therapy to better guide the procedure. In order to evaluate the SNR, a 4-channel array is fabricated using the optimized material stack of PEEK, PTFE, and silver ink. The SNR of the array is compared to that of the currently used body coil of a 3 T scanner on a gel phantom inside the head transducer. Figure 4A illustrates the positioning of the printed array wrapped around the gel phantom and submerged inside the head transducer to characterize the SNR. The SNR across the center of the phantom - highlighted in Fig. 4B,C - shows that the array presents 5 times the SNR at the surface of the phantom when compared to the currently used body coil. The asymmetry seen in the coil sensitivity pattern is due to the coil size and the placement on the phantom. At the center of the phantom, where a MRgFUS procedure is most likely to occur, the array displayed twice the SNR when compared to the body coil. This reduction in the center could be improved by better-optimized element size and placement, but it is beyond the scope of the work presented here. The array also shows more localized sensitivity to the surrounding water and transducer than the body coil, offering additional opportunities to decrease the field of view and shorten the scan time.

To show the clinical SNR gains that a printed coil array can provide, breath-hold abdominal images are acquired with an 8-channel coil array wrapped around the abdomen of a volunteer. The comparison between the abdominal images from the body coil and the transparent arrays in Fig. 4D shows that it is possible to obtain images with more detailed liver and stomach regions when using the printed array. Similar to the phantom testing results, the 8-channel array showed the highest SNR at the surface of the volunteer and presents double the SNR in the center of the body. The increased detail would be valuable during treatments and planning surgeries. In addition to the observed SNR benefit, the multichannel array is also able to perform parallel imaging acceleration from the additional channels enabling faster image acquisition^15^.

The array and body coil are used to track ultrasonic heating inside a gel phantom. Figure 4E shows axial and coronal slices of the maximum heating point for each of these experiments. The heating occurs in the center of the phantom where the 8-channel printed array has slightly more than double the SNR of the body coil. In regions of the phantom that did not see any heating, the standard deviation of temperature estimated was ± 0.84 °C from images obtained with the body coil and ± 0.19 °C in images from the array. As a result, in both the coronal and axial slices of the heating profile, the coil array provides clearer heating profiles. This is more evident in the coronal profile where the printed array easily shows the side lobes of the heating from the focal point, while the body coil only provides a faint outline of the total profile.

### Array System Level Integration with MRgFUS System on Phantoms and *Ex-vivo* Tissue

**Figure 5:**
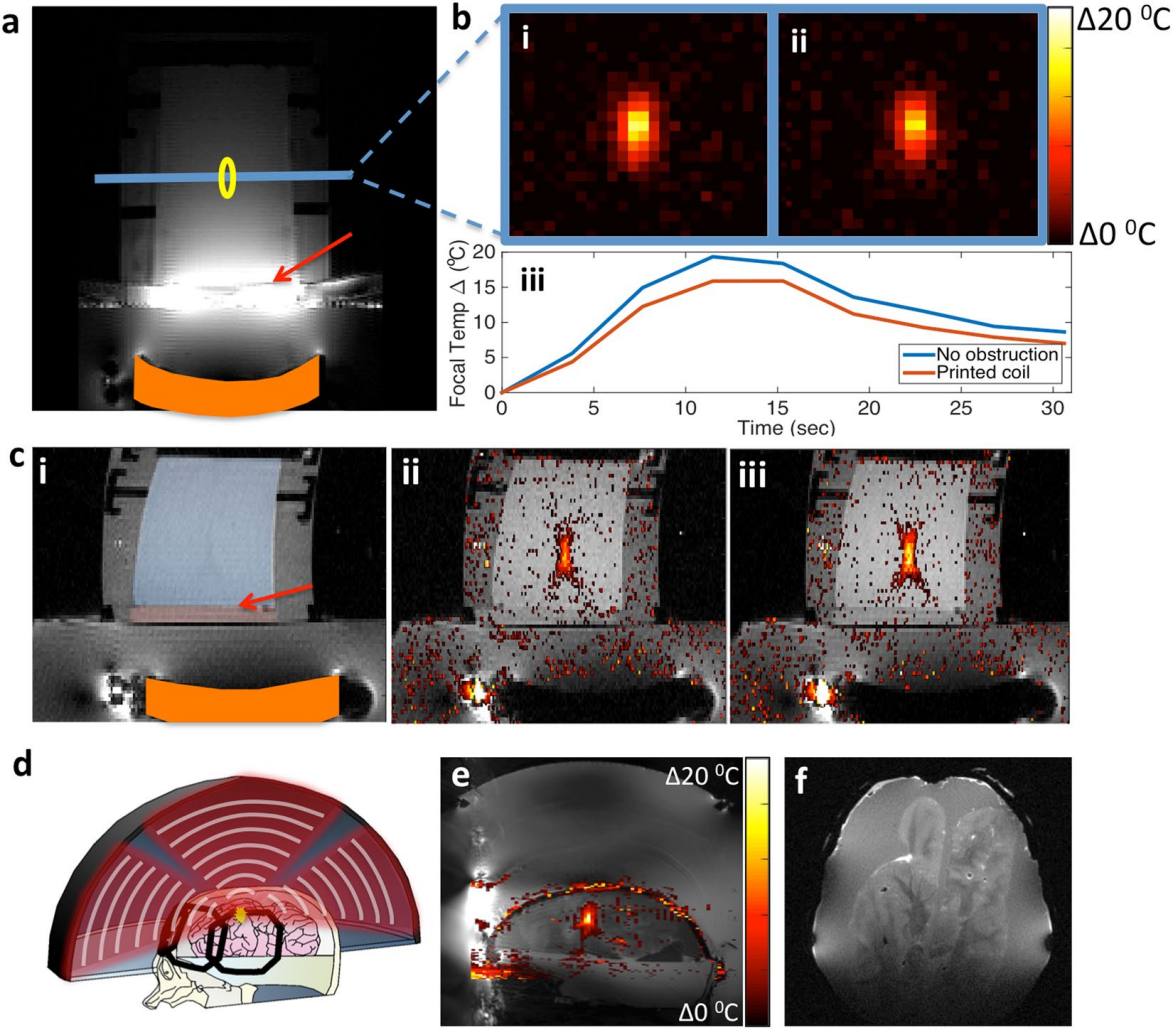
Printed array in MRgFUS heating and imaging. (**a**) Scan of gel phantom on a fibroid transducer using printed array. Red arrow shows the location of array, orange highlights location of the transducer, blue line shows location of slice used to track heating, and yellow shows the approximate location of heating. (**b**) Heating point inside of phantom (i) without and (ii) with the array present. Heating is tracked with body coil in both cases for maintain the same conditions for comparison. (iii) Plot showing temperature change at the focal point vs. time. (**c**) (i) Scan of gel phantom on fibroid transducer highlighting placement of coil (arrow) between top (blue) and bottom (red) gel phantoms. Thermometry scans (ii) with coil placed between gel phantoms and (iii) without. (**d**) Illustration showing the printed array wrapped around the skull and brain phantom containing a bovine brain submerged in water inside the head transducer. (**e**) Sagittal image of the brain phantom with overlaid heating map tracked with the 4-channel array. Hot spot near center of phantom is the focal point of transducer. Areas inside plastic and air regions of the phantom have large phase shift and appear as random hotspots in image. Regions of the brain near the front of the head phantom where the coil array has higher SNR and less phase error compared to the back of the head. (**f**) Axial scan of the skull and brain phantom showing anatomical image quality from the 4-channel array.

The acoustic attenuation of the coil is measured on the scanner by heating an area inside a homogeneous gel phantom to produce approximately 20 °C of temperature rise. For clarity, the annotated scan in Fig. 5A illustrates how the coil is placed in-between the transducer and the phantom during these experiments. The temperature increase is tracked with the body coil of a 3 T scanner with and without the array to maintain the measurement consistency. Figure 5B shows examples of the temperature maps taken with the body coil without and with the 4-channel array present. When the 4-channel array is placed between the transducer and the phantom, 83 ± 3% of the temperature rise is measured without any noticeable beam distortion. This value matches those seen in the water bath testing along with the acoustic modeling. This 17% attenuation is considerably smaller than the attenuation due to the skull, which is approximately 70%^34^. This attenuation would be much smaller on the 650 kHz head system as suggested by the water bath testing, however the low image SNR from the body coil did not allow precise temperature measurement for this comparison. The transmission of the coil array could be improved if the centers of the coils are removed, but our testing accurately captures the worse case attenuation.

In order to verify that the coils are not absorbing any significant amount of energy that could pose a risk to any nearby tissue, an additional 1.5 cm thick agar gel disk is placed underneath the coil completely surrounding it in material that MR thermometry could be used to measure temperature increase. Next, 54 W of acoustic power is transmitted though the gel stack for 10 seconds with and without the coil present to see if there is any measureable increase temperature near the coil. Figure 5C shows the thermometry maps inside the gel phantoms with and without the coil present. There is no measurable increase in temperature at or near the coil suggesting that it did not absorb any significant amount of power during the sonication. Afterwards, a second sonication was performed at much lower power to record the amount of reflection seen at the transducer. The amount of reflected signal seen at the transducer was 13% higher with the coil present. Additional information about the reflection experiment is in the extended methods. This measurement is not directly relatable to how much power is reflected by the coil since not all the reflected energy was captured by the transducer and the signal-to-pressure conversion factor is not well characterized for this analysis, but the increase suggests that the power lost is reflected by the coil water interface rather than absorbed by coil materials.

To demonstrate the proof-of-concept of all system elements together, a 4-channel array is used to track the heating of brain tissue inside the head transducer. A 3D printed ABS plastic skull mimics bone containing an *ex-vivo* bovine brain suspended in a gel as described in Menkiou *et al*.^35^ Fig. 5D illustrates the positioning of the 4-channel array on the skull phantom while it is heated inside a head transducer. The temperature map obtained is overlaid on the anatomy scan of the bovine brain in Fig. 5E. The temperature map in Fig. 5E is similar to the heating profile shown in Fig. 4E, indicating there is not significant distortion or attenuation due to the array. Similar to the phantom scans, SNR in the heating region is twice as high as that given by the body coil. Additionally, a high-resolution scan of the brain phantom is taken inside the transducer, shown in Fig. 5F. This scan shows that the highest SNR is at the front of the brain near the coil and slowly drops off towards the back of the head where there is no array. Using additional arrays can increase coverage, but it is beyond the capability of our current experimental setup. Overall the array shows up to 5 times the SNR at the surface of the body near the coil than the currently used body coil while tracking the heating point inside the skull without significantly attenuating or visibly distorting the acoustic power. For procedures done in the center of the body, the array presented here shows SNR twice as high as the body coil.

Our array outperforms the currently used body coil while tracking the heating point inside the skull without significantly attenuating or visibly distorting the acoustic power.

## Discussion

The optimized printed materials do not suffer from water stability problems and have high acoustic transmission making them uniquely suited for MRgFUS coils. Our printed coils show high SNR in phantoms, *ex-vivo* tissue, and volunteers without significantly interfering with the operation of the MRgFUS system. The increased SNR from this coil array allows more precise estimation of the temperature increase, particularly near the focal point.

With the advances presented in this work, coils designed for MRgFUS can now utilize the current state of the art array designs without the restriction imposed by the position of the ultrasonic transducer. The high SNR offered by these designs provides better resolution, allows for more intricate sequences to be run, and enables faster acquisition of heat maps to monitor treatment. This work can bring MRgFUS the powerful imaging tools that physicians and researchers are accustomed to have in diagnostic imaging, enabling new methods of treatment for this highly versatile technique.

## Methods

### Coil Array Fabrication

Octagonal coils 8.75 cm in diameter are screen printed onto plastic substrates using a conductive silver ink (Dupont 5064 H) patterned through a 165 count stainless steel mesh (Meshtec). Individual array coils are tuned to 127.73 MHz by changing the area of the in-series capacitors. Coils are then laminated in a PTFE film (Professional Plastics) for water protection, abrasion resistance, and volunteer safety. Coils are connected to a non-printed interface board that contains an inductor and diode to block the coil during the high power RF transmit. A half wavelength long piece of RG-316 non-magnetic cable then connects to a box containing preamplifiers (MR Solutions) which then connects to the scanner.

### Water Stability Characterization

Several plastic substrates are selected based on their known water absorption data, mechanical/thermal stability, and availability in thicknesses less than 150 μm. The copper/acrylic testing rig simulates our 8.75 cm diameter coil size using 35 μm thick copper strips between two 5 mm thick acrylic sheets clamping the substrate in the middle. The area of the copper strips is trimmed so that the coil structure resonated at the Larmor frequency of our 3 T scanner (127.73 MHz). Coils are wiped dry after being pulled out of the water before being placed back into the testing rig for measurement.

DuPont 5064 H silver ink is chosen for the conductive portions of the coil based on its previous use in printed MRI coils^28^. To characterize the stability of the conductive traces in water, several samples ranging from 3–28 μm of DuPont 5064 H are measured on a 4-point probe to determine bulk resistivity before being submerged in 20 °C deionized water for 24 hours. Then, the traces are wiped dry and re-measured on the 4-point probe to characterize any change in the conductivity. Additionally, the film surface roughness is characterized before and after water submersion on a profilometer to determine if there is any difference in film topography.

### Acoustic Characterization

To evaluate the materials, test films are placed in a deionized water bath between an ultrasonic transducer (Olympus V303-SU) and a calibrated hydrophone (Onda HGL-0400 capsule hydrophone with AH-2020 20 dB preamplifier) placed 2.54 cm apart. The tank is sized to be sufficiently large compared to the wavelength of the acoustic energy (45 × 30 × 30 cm) and lined with sound attenuating foam in order to minimize reflections of sound waves off the sidewalls. All values are normalized to the acoustic pressure when no obstruction is present.

A 1-D wave model is used to characterize the acoustic properties of the printed coil materials because our printed films were thin compared to the focal length of the transducer. A non-linear least squares fit is used to estimate the acoustic impedance using an acoustic transmission line model. An in depth explanation of this method is described in Kino, *et al*.^17^ and is summarized in the extended methods. The model was fit to our data using a trust-region-reflective non-linear least squares fit^36^.

### Imaging Parameters

The SNR of our array is compared to the SNR of the traditionally used body coil of a 3 T scanner (General Electric 3 T Discovery MR750) on a gel phantom inside the head transducer (Insightec Exablate 4000).

An ultra fast gradient echo scan with flip angle of 30°, encode time of 12.7 ms, readout time of 25.6 ms and 1 average sequence is chosen as a representative scan of what would be used in a temperature map to compare SNR.

A gradient echo sequence with flip angle of 20°, encode time of 4 ms, readout time of 8.6 ms, 1 average is used to acquire images with both the body coil and the printed array. Images of the volunteer are acquired on the same system described previously, but with the transducer unpowered for safety. The coil array was offset from the volunteer by 4 mm to reduce capacitive coupling. All volunteer imaging was performed with their informed consent in accordance with internal review board (IRB) approval at Stanford and UC Berkeley.

### Phantom and *Ex-Vivo* Tissue Heating

Gel phantoms (Insightec Gel Phantom) have 60 W of acoustic power applied for 10 seconds at 650 KHz inside the head transducer. Using the in-table transducer (Insightec ExAblate 2100) phantoms receive 54 W of acoustic energy at 1 MHz for 10 seconds for an approximate temperature rise of 20 °C. An axial slice of the beam is prescribed to map the temperature every 3.4 seconds. The maximum temperature recorded is used as the benchmark for comparison. The acoustic power is applied at the same time to ensure accurate capture of the maximum heating point. To prevent the focal spot of the transducer from only being partially captured by the single slice, the temperature increase is measured 10 times, each point evenly spaced along 10 mm of the focal point of the transducer. After scanning, the complex image data was reconstructed to show the temperature increase by measuring the phase difference^2^.

The 3D printed ABS plastic skull mimics bone containing an *ex-vivo* bovine brain suspended in a gel of 2% agar, 1.2% silica, and 25% evaporated milk as described in Menkiou *et al*.^35^. A thin latex membrane is stretched around the entire phantom to prevent animal tissue from contacting the clinical system. The phantom is mounted to the patient table of the head transducer and scanned with an ultra fast gradient echo that had a flip angle of 30°, TE of 12.8 ms, TR of 25.7 ms, 1 average. An imaging slice 34 × 34 cm and 3 mm thick with 256 frequency encodes and 128 phase encodes is taken every 3.4 seconds to track heating. A head transducer (Insightec Exablate 4000) applied 200 W of acoustic power to the targeted area for 10 seconds. The heating map is overlaid onto an anatomy scan obtained using a fast relaxation fast spin echo with TE of 100.7 ms, TR of 4565 ms, FA of 111°, and 2.5 averages. Each slice is 34 × 34 cm and 2 mm thick.

## Electronic supplementary material

Extended Methods
