# Supplementary material for: Printed Receive Coils with High Acoustic Transparency for Magnetic Resonance Guided Focused Ultrasound
Source: Sci Rep. 2018 Feb 21;8:3392. doi: 10.1038/s41598-018-21687-1 (PMC5821831; doi:10.1038/s41598-018-21687-1)
Supplement: Supplementary file 1 — Extended Methods [file 41598_2018_21687_MOESM1_ESM.doc]

**Extended Methods for:**

**Printed Receive Coils with High Acoustic Transparency for Magnetic Resonance Guided Focused Ultrasound**

Joseph Corea^1^, Patrick Ye^2^, Dongjin Seo^1^, Kim Butts-Pauly^2^, Ana Claudia Arias^1^ and Michael Lustig^1^

^1^Electrical Engineering and Computer Sciences, University of California Berkeley, CA 94720

^2^Radiology, Stanford University, Stanford, CA 94305

*Acoustic model*

If the substrate is thin compared to the focal distance of the transducer, then it is possible to model the system as a 1D wave traveling through regions of different acoustic impedance. Since the vast majority of power is lost due to reflections and wave effects, this 1D system can be described by the transmission line equations used for electromagnetic waves to predict the amount of acoustic power seen at the load. An in depth explanation of this relationship is described in Kino, et al ^17^, but is described here for clarity. More complex models that take loss from attenuation in the bulk due to heat generation are available, however, the films of interest here are very thin when compared to the typical attenuation constant and do not lose much energy to heat.

The specific acoustic impedance (Z_0_) in kg/(m^2^s) can be calculated by the equation that relates the material’s speed of sound (c) in m/s and the density (ρ) in kg/m^3^ (equation 1).


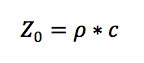
 (1)

To calculate the amount of power dissipated in the film and load, an equivalent circuit can be setup like the one shown in supporting Fig. S1.


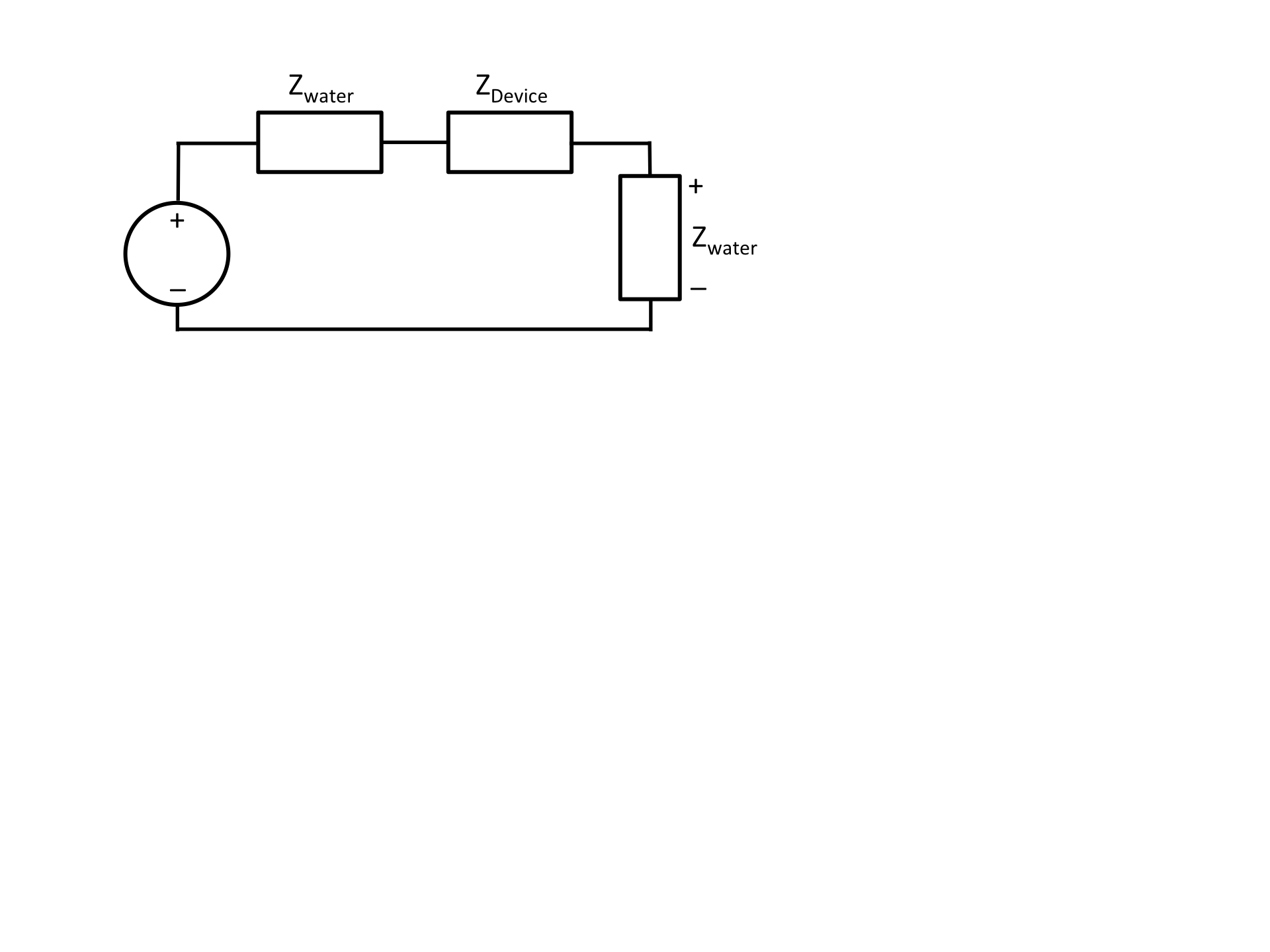


**Fig. S1:** Equivalent circuit used to calculate power dissipated in load (hydrophone). In this model it is assumed both the transducer and hydrophone are perfectly matched to water.

The current through the circuit can be calculated using ohms law (equation 2, where the amplitude of the acoustic pressure is V) once all impedances are known, however, the impedance at any point in the circuit is dependent on the impedance it is loaded with (i.e., load impedance, Z_L_). The impedance looking into the load (Z_in_) can be calculated using equation 3 where L is the thickness of the film and ω is the angular frequency.


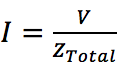
 (2)


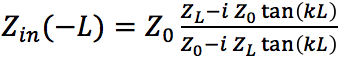
 where:
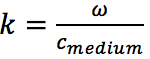
 (3)

Once the current in the circuit is known, the power dissipated in the load (P) can be found using equation 4.


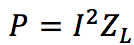
 (4)

In our analysis, the absolute magnitude of the acoustic pressure was not known, so all the measurements, including those predicted in the model, were normalized to the pressure seen at the load when no film was present.

The speed of sound, density, and acoustic impedance values used in our model are shown in supporting table ST1^31,32,35,36^.

Table ST1: Values used in acoustic impedance analysis

| Material | Density (kg/m^3^) | Speed of Sound  (m/s) | Acoustic Impedance  (MRayl) |
| --- | --- | --- | --- |
| PEEK^36^ | 1260 | 2536 | 3.16 |
| PTFE^32^ | 2149 | 1390 | 2.99 |
| Water^31^ | 1000 | 1482 | 1.48 |

**Dielectric Water Stability**

Several plastic substrates are selected based on their known water absorption data, mechanical/thermal stability, and availability in thicknesses less than 150 μm. The copper/acrylic testing rig simulates our 8.75 cm diameter coil size using 35 μm thick copper strips between two 5 mm thick acrylic sheets clamping the substrate in the middle. The area of the copper strips is trimmed so that the coil structure resonated at the Larmor frequency of our 3T scanner (123.3 MHz). Coils are wiped dry after being pulled out of the water before being placed back into the testing rig for measurement.

**Conductor Water Stability**

DuPont 5064H silver ink is chosen for the conductive portions of the coil based on its previous use in printed MRI coils^28^. To characterize the stability of the conductive traces in water, several samples ranging from 3-28 μm of DuPont 5064H are measured on a 4-point probe to determine bulk resistivity before being submerged in 20 °C deionized water for 24 hours. Then, the traces are wiped dry and re-measured on the 4-point probe to characterize any change in the conductivity. Additionally, the film surface roughness is characterized before and after water submersion on a profilometer to determine if there is any difference in film topography.

After submersion, the samples made of the DuPont 5064H silver ink did not experience a significant change in resistivity; showing resistivity of 16±2 μohm-cm before and after submersion. Furthermore, the surface roughness of the ink did not change, maintaining a root mean squared (RMS) surface roughness of 1.3±0.2 μm both times. From these results, the stability of DuPont 5064H is sufficient for use in an MRgFUS application.

**Acoustic Transmission Testing**

To evaluate the materials, test films are placed in a deionized water bath between an ultrasonic transducer (Olympus V303-SU) and a calibrated hydrophone (Onda HGL-0400 capsule hydrophone with AH-2020 20 dB preamplifier) placed 2.54 cm apart. The tank is sized to be sufficiently large compared to the wavelength of the acoustic energy (45x30x30 cm) and lined with sound attenuating foam in order to minimize reflections of sound waves off the sidewalls. All values are normalized to the acoustic pressure when no obstruction is present. The acoustic measurements are averaged 2048 times to reduce noise. Each trial is repeated 3 times to further reduce the measurement error. The experimental setup was characterized to have a relative error of 5% by measuring the relative intensity of the focal spot by the hydrophone with no obstruction several times over several different measurement sessions. In our analysis, the absolute magnitude of the acoustic pressure was not known, therefore all the measurements, including those predicted in the model, were normalized to the pressure seen by the hydrophone when no film was present.

Silver ink films thicker than 28 μm are difficult to produce with the screen printer, so to obtain films thicker than 28 μm for the acoustic characterization, a blade coater is used to print 17, 24, 38, and 56 μm thick films. Films thicker than 56 μm are extremely non-uniform, showing large deviations in thickness across the film and are not characterized.

**Encapsulation Testing**

The electrical isolation of each encapsulation was tested by applying it over a conductive printed trace of DuPont 5064H on 76μm PEEK substrate. Then the samples are then submerged in a 1 molar solution of salt water that is biased to 10,000V. If any current through the film is observed, then the film is not suitable to protect the patient from the DC electrical contact to the coil and was not used.

To characterize how well the barrier films adhered to the surface of the PEEK, a piece of adhesive tape was pressed in contact with the film and then quickly removed. If any of the barrier material delaminated from the substrate, then the films were not appropriate for coil construction. A summary of the acoustic, electrical breakdown testing, and adhesion testing is shown in Table ST2.

**Table ST2: Acoustic, breakdown, and adhesion data for various encapsulation me**thods.

| Encapsulation | Thickness | Acoustic Transmission (Pressure) | 10,000V breakdown test | Tape test |
| --- | --- | --- | --- | --- |
| PTFE film | 76 μm | 97.8 % | Pass | Pass |
| Epo-Tek Flexible Epoxy | 100 μm | 94.2 % | Fail | Pass |
| FEP film | 90 μm | 94.0 % | Pass | Pass |
| Polyimide film | 50 μm | 93.7 % | Fail* | Pass |
| PMMA Paint | 10 μm | 91.9 % | Fail | Fail |
| PMMA Sealer | 10 μm | 91.7 % | Fail | Fail |
| Rubberized coating | 50 μm | 91.6 % | Fail | Pass |
| PET film | 50 μm | 87.3 % | Pass | Pass |
| PEEK film | 50 μm | 82.3 % | Pass | Pass |
| PTFE film | 125 μm | 77.5 % | Pass | Pass |

*adhesive failed

Based on the initial absorption data shown in table 2, we found that a 76 μm film of PTFE with an acrylic adhesive was able to easily stop water transmission and adhere to our coil without delaminating, while passing the highest amount (97.8%) of the initial acoustic pressure. This substrate provided high breakdown strength, easily holding the 10,000V DC bias placed across it. This film provided excellent mechanical and electrical encapsulation that could be further optimized.

The PTFE encapsulation material is evaluated over 600 kHz to 1.4 MHz in steps of 5 kHz. Scanning over a 20x20 mm area created a 2D map of transmitted power. The porcelain capacitors (ATC series B) on 35 μm copper coated 1 mm thick fiberglass board. In order to compare it with the traditionally used coil materials, a 2 mm thick piece of acrylic sheeting was characterized to show the attenuation that could occur from typical array packaging.

**Scan Settings**

The SNR of our array is compared to the SNR of the traditionally used body coil of a 3T scanner (General Electric 3T Discovery MR750) on a gel phantom inside the head transducer (Insightec Exablate 4000).

An ultra fast gradient echo scan with flip angle of 30^o^, encode time of 12.7 ms, readout time of 25.6 ms and 1 average sequence is chosen as a representative scan of what would be used in a temperature map for the SNR comparison.

A gradient echo sequence with flip angle of 20^o^, encode time of 4 ms, readout time of 8.6 ms, 1 average is used to acquire images with both the body coil and the printed array. Images of the volunteer are acquired on the same system described previously, but with the transducer unpowered for safety. The coil array was offset from the volunteer by 4 mm to reduce capacitive coupling. All volunteer imaging was performed with internal review board (IRB) consent.

**Phantom Heating**

Gel phantoms (Insightec Gel Phantom) have 60 W of acoustic power applied for 10 seconds at 650 KHz inside the head transducer. Using the in-table transducer (Insightec ExAblate 2100) phantoms receive 54W of acoustic energy at 1 MHz for 10 seconds for an approximate temperature rise of 20 °C.

An axial slice of the beam is prescribed to map the temperature every 3.4 seconds. To prevent the focal spot of the transducer from only being partially captured by the single slice, the temperature increase is measured 10 times, each point evenly spaced along 10 mm of the focal point of the transducer. After scanning, the complex image data was reconstructed to show the temperature increase by measuring the phase difference^2^. The maximum temperature recorded is used as the benchmark for comparison. The acoustic power is applied at the same time to ensure accurate capture of the maximum heating point.

**Figure S2:** Bar graph of average reflected voltage signal seen at fibroid transducer over 208 elements. Error bars represent standard deviation.

To measure the reflected signal at the fibroid transducer when the coil was placed in the heating phantom a low power pulse was sent at the phantom. Figure S2 shows the average peak-to-peak voltage of the reflection signal averaged across all 208 elements of the transducer. A paired student t-test was used to analyze the statistical significance of the difference across all 208 elements finding a statistical significance of p<1e-5 As is shown, there is a 13% increase in the signal when the coil is inside the heating gel over when it is empty.

The 3D printed ABS plastic skull mimics bone containing an ex vivo bovine brain suspended in a gel of 2% agar, 1.2 % silica, and 25 % evaporated milk as described in Menkiou et al.^34^. A thin latex membrane is stretched around the entire phantom to prevent animal tissue from contacting the clinical system. The phantom is mounted to the patient table of the head transducer and scanned with an ultra fast gradient echo that had a flip angle of 30^o^, TE of 12.8 ms, TR of 25.7 ms, 1 average. An imaging slice 34x34 cm and 3 mm thick with 256 frequency encodes and 128 phase encodes is taken every 3.4 seconds to track heating. The head transducer applied 200 W of acoustic power to the targeted area for 10 seconds. The heating map is overlaid onto an anatomy scan obtained using a fast relaxation fast spin echo with TE of 100.7 ms, TR of 4565 ms, FA of 111^o^, and 2.5 averages. Each slice is 34x34 cm and 2 mm thick.
